# Use of Chloroform in Retention of Urine

**Published:** 1857-12

**Authors:** 


*Use of Chloroform in Retention of Urine.*—An intemperate cabman, aged 52, was admitted into a medical ward at Guy's, a few days ago, on account of chest symptoms. It appeared that he had had gonorrhœa twelve years before, and had ever since had more or less difficulty in passing his water. After having been in the hospital nearly three weeks, he was seized with retention of urine. The dressers and house surgeon made patient and repeated attempts to pass a catheter, but without result. There was little doubt that the stricture was a permanent one, which had been closed by inflammation. In February the retention had become complete for two days; the symptoms were becoming very urgent, and Mr. Cooper Forster was accordingly called to see him. Opium had been most freely given. Having failed in persevering attempts to introduce a new No. 2 catheter, Mr. Forster determined to administer chloroform, and, if needful, puncture the bladder by the rectum. When completely insensible, another trial was made with a No. 3, which now passed most readily. We cite this case as important, because it proves beyond dispute, the influence of the anæsthetic state in relaxing an otherwise impermeable stricture. An opiate treatment had been fairly tried before, and had failed, and the catheter had also been found useless in the hands of several well-practiced surgeons. The plan of administering chloroform in cases of obstinate stricture and retention, is one in wide use, both in hospital and private practice; but, as it is not yet in such general favor as it deserves to be, we have thought that so pointed an example of its advantages might be worth bringing before our readers.—*Medical Times & Gaz.*

We add to the above the history of a case which was kindly furnished us by Prof. Hamilton, which very nearly resembles the above, and which seems to show that the difficulty of passing an instrument, often attributed to inflammation and consequent swelling of the parts, is really due to spasm, as in Dr. Hamilton's case, "at the moment of the most extreme prostration the instrument fell into the bladder by its own weight." F.

"George Williams, brakesman, State Line R. R., was severely injured in a collision, Oct. 8th, (Thursday); on the 9th Dr. Hamilton was able to introduce a catheter to relieve his bladder, but on the morning of the 10th, he could not, owing to the swelling in the perineum and spasm in the urethra. Repeated and prolonged attempts were made on the 10th and 11th, and on the morning of the 12th, at the suggestion of Dr. Flint, chloroform was administered until complete anæsthesia was produced, and at the moment of the most extreme prostration the instrument fell into the bladder by its own weight. He had retained his urine sixty hours."

